# Building Resilience to Climate Change: Pilot Evaluation of the Impact of India's First Heat Action Plan on All-Cause Mortality

**DOI:** 10.1155/2018/7973519

**Published:** 2018-11-01

**Authors:** Jeremy J. Hess, Sathish LM, Kim Knowlton, Shubhayu Saha, Priya Dutta, Parthasarathi Ganguly, Abhiyant Tiwari, Anjali Jaiswal, Perry Sheffield, Jayanta Sarkar, S. C. Bhan, Amit Begda, Tejas Shah, Bhavin Solanki, Dileep Mavalankar

**Affiliations:** ^1^University of Washington, Seattle, WA, USA; ^2^Indian Institute of Public Health, Gandhinagar, Ahmedabad, India; ^3^Natural Resources Defense Council, New York, NY, USA; ^4^Emory University, Atlanta, GA, USA; ^5^Icahn School of Medicine at Mount Sinai, New York, NY, USA; ^6^Indian Meteorological Department, Gujarat, India; ^7^Indian Meteorological Department, Delhi, India; ^8^Ahmedabad Municipal Corporation, Ahmedabad, India

## Abstract

**Background:**

Ahmedabad implemented South Asia's first heat action plan (HAP) after a 2010 heatwave. This study evaluates the HAP's impact on all-cause mortality in 2014–2015 relative to a 2007–2010 baseline.

**Methods:**

We analyzed daily maximum temperature (*T*_max_)-mortality relationships before and after HAP. We estimated rate ratios (RRs) for daily mortality using distributed lag nonlinear models and mortality incidence rates (IRs) for HAP warning days, comparing pre- and post-HAP periods, and calculated incidence rate ratios (IRRs). We estimated the number of deaths avoided after HAP implementation using pre- and post-HAP IRs.

**Results:**

The maximum pre-HAP RR was 2.34 (95%CI 1.98–2.76) at 47°C (lag 0), and the maximum post-HAP RR was 1.25 (1.02–1.53) estimated at 47°C (lag 0). Post-to-pre-HAP nonlagged mortality IRR for *T*_max_ over 40°C was 0.95 (0.73–1.22) and 0.73 (0.29–1.81) for *T*_max_ over 45°C. An estimated 1,190 (95%CI 162–2,218) average annualized deaths were avoided in the post-HAP period.

**Conclusion:**

Extreme heat and HAP warnings after implementation were associated with decreased summertime all-cause mortality rates, with largest declines at highest temperatures. Ahmedabad's plan can serve as a guide for other cities attempting to increase resilience to extreme heat.

## 1. Introduction

The dangers of extreme heat to health are extensively documented [1–5]. Globally, extreme heat events have become more frequent and intense over recent decades [6], and several extreme heat events have been fully attributed to climate change [7]. These trends are expected to accelerate, with an average projected global temperature increase of at least 0.2°C per decade by 2030 and as much as 4.8°C by the end of the century relative to a 1986–2005 baseline without aggressive mitigation of greenhouse gas emissions [8]. There is high confidence that this warming may have significant adverse health impacts [9] and that various preparedness strategies and early warning systems will need to be a part of climate change adaptation planning [9, 10]. In many instances, heat is already a threat to health, and current efforts to promote resilience to extreme heat can facilitate climate change adaptation [11].

Low- and middle-income countries are particularly vulnerable to extreme events including heat waves and suffer the greatest losses from extreme weather [12]. South Asia is one of the highest-risk areas for extreme heat [13]. There are known associations between heat exposure and all-cause mortality in India, with evidence of a threshold at 40°C above which mortality increases [14]. Heat waves have killed many thousands in the region in recent years [15–17], though data limitations constrain accurate estimates of true health effects. Vulnerability to extreme heat is high among residents of informal settlements (housing on land to which residents have no legal claim) [18] and workers [19]. While air conditioning, one of the most effective protective measures against heat [20], is becoming more prevalent in the region; it remains unavailable to many due to cost, and infrastructure failures make it unreliable [21]. Much of the population in South Asia remains highly exposed and susceptible to the adverse effects of extreme heat.

Exposure to extreme heat in South Asia is likely to increase as extreme heat events continue to worsen with climate change. India has experienced warming consistent with the globally observed trend [22], and projections suggest that heat waves there will become progressively more frequent and severe over the course of the 21^st^ century [23]. Even in highly protected settings where the population has adapted to warm temperatures over time, climate change is projected to result in significant excess mortality [24], and presumably, impacts will be more significant in the highly exposed population of South Asia compared to populations in higher income areas.

Ahmedabad, the largest city in the Indian state of Gujarat and one of India's fastest growing, is a useful case study for studying heat health impacts and prospects for improving resilience. Like other areas, Gujarat has warmed, with average *T*_max_ increasing by 0.11°C from 1969 to 2005 [25]. In 2010, Ahmedabad experienced a heat wave with a *T*_max_ of 46.8°C, and then the highest in nearly a century. All-cause mortality rose sharply, and there was an excess 1,344 all-cause deaths in May 2010 alone, a 43.1% increase over the baseline mortality rate [26]. This event put heat stress in the headlines and made heat preparedness a priority for the city government, the Ahmedabad Municipal Corporation (AMC).

Based on the spike in mortality during the 2010 heat wave, the AMC partnered with the Public Health Foundation of India, the Indian Institute of Public Health-Gandhinagar, the Natural Resources Defense Council, and a coalition of international partners to develop a heat action plan (HAP) for Ahmedabad. The team relied on best practices [27, 28] to develop the system and selected interventions that had been cited as effective in prior evaluation studies [29, 30]. The Ahmedabad HAP, described in detail elsewhere [31], issued progressively more urgent warnings and coordinated significant interagency activities when *T*_max_ was forecasted to exceed 41°C. The HAP was piloted in 2013 and fully implemented in 2014. It was immediately put to the test, as the summers of 2014 and 2015 were unusually hot, although the summer daily maximum temperature profiles varied.

The objective of this study is to compare associations between extreme ambient heat and all-cause mortality before and after the HAP was implemented to quantify its effect, and add to the evidence base by expanding the range of settings in which HAP efficacy has been documented.

## 2. Materials and Methods

### 2.1. Intervention

The Ahmedabad HAP has three key elements: community outreach to build awareness, a probabilistic forecast and early warning system [32], and health care capacity building. There are multiple warning levels (yellow, orange, and red) depending on forecasted temperatures, with each warning level corresponding to increasing levels of outreach, preparatory activity, and response. According to best practices, warning thresholds were developed based on retrospective epidemiological analyses of health-relevant temperature thresholds [26, 31]. *T*_max_ was used as the exposure metric given the low historical summertime humidity in the region. Alerts were structured as follows: no alert was issued for forecasted *T*_max_ of up to 40°C; a yellow alert was issued for forecasted *T*_max_ 41–42.9°C; an orange alert was issued for forecasted *T*_max_ 43–44.9°C; and a red alert was issued for forecasted *T*_max_ of 45°C or higher. Activities were coordinated through a nodal officer in the health department of the city government. Alert activities are described fully in previous work [31].

### 2.2. Evaluation Approach

Data were collected in 2016 and analyzed from 2016 to 2018. We chose to use 2007–2010 as the baseline period and 2014–2015 as the postintervention period because HAP planning and implementation occurred from 2011 to 2013, with full implementation starting in 2014. Given Ahmedabad's rapid growth, we determined that a short baseline would be more comparable and a longer baseline period would result in many unmeasured factors that might be less comparable. We used temperature, mortality, and population data (described below) to analyze how the relationship between *T*_max_ and all-cause mortality changed after the HAP was implemented. We performed a descriptive analysis of daily *T*_max_ distributions before and after the HAP to compare the respective heat seasons. We conducted a distributed lag analysis of the relationship between *T*_max_ and all-cause mortality to evaluate how relative risk lag structures changed after the intervention. To incorporate changes related to population growth, we calculated mortality incidence using year-specific population estimates for all days with forecasted *T*_max_ over 40°C (i.e., any heat alert) and for specific heat alert strata (i.e., yellow, orange, and red alert days, respectively). Using these results, we used pre-HAP IRs to calculate expected post-HAP mortality and compared this with observed mortality for days with forecasted *T*_max_ over 40°C to calculate a postintervention mortality difference.

### 2.3. Data Sources

#### 2.3.1. Temperature Data

Daily temperatures were obtained from the Meteorological Aviation Report (METAR) system, which reports data from an automated weather observing system (AWOS) weather station (METAR station identification number: VAAH 040710Z 01003KT 4000 DU FEW080 40/21 Q1006 NOSIG) located at Ahmedabad's Sardar Vallabai Patel International Airport. We collected hourly temperature data from 1 April to 30 June for 2007–2010 and 2014–15, respectively, and identified *T*_max_ as the highest value in each daily sample downloaded from the web-based portal.

#### 2.3.2. Mortality Data

The AMC assembles daily mortality data for the city using death reports from hospitals, crematoria, burial grounds, and other sources. Death registration and a death certificate are required prior to cremation or burial. The data from all sources are compiled by the AMC Registrar of Births and Deaths within three weeks and typically finalized within several months. We obtained daily death data for each year in the study period from Registrar of Births and Deaths office of AMC.

#### 2.3.3. Population Data

India performs a population census every decade; the last census occurred in 2011. Ahmedabad city's 2011 population was 5,577,940, and its 2001 population was 3,520,085 [33]. We used these two census counts and assumed compounding growth [34] to calculate a compound average annual growth rate of 4.71%. This growth rate and the 2001 and 2011 census counts were used to interpolate annual population estimates. This rate was used to generate population estimates for the intercensal years during the study period. Population estimates were 4,639,867 for 2007, 4,858,449 for 2008, 5,087,328 for 2009, 5,326,989 for 2010, 6,403,981 for 2014, and 6,705,669 for 2015.

### 2.4. Data Preparation

We used the annual population estimates to generate daily all-cause mortality rates (deaths per 100,000 population) from crude daily mortality data. There were no missing days of data.

### 2.5. Analytical Approach

Analyses were performed in Microsoft Excel 2010 (Redmond, WA) and *R* version 3.4.0.

#### 2.5.1. Descriptive Analysis

To evaluate the intensity of heat exposure across the study period, we plotted curves for daily *T*_max_ for the years 2007–2010 and 2014–2015 and visually inspected the two periods to determine how heat exposure was distributed over each.

#### 2.5.2. Distributed Lag Nonlinear Modeling of Temperature-Mortality Relationship

To estimate changes in relative risk of mortality associated with heat exposure, we used the *R* package dlnm version 2.3.4 [35] to estimate mortality rate ratios (RRs) for daily *T*_max_ at lags up to 5 days, estimating mortality rates for each temperature relative to rates at 40°C. We derived the model using 2007–10 data and then applied the same model to data from 2014 to 2015. A priori, we modeled the cross-basis function for temperature as natural cubic spline with 5 knots, two in the boundaries and remaining knots at 40°C, 42°C and 45°C, respectively. We used 3 equally spaced knots in the log-scale as the lag response function over lags 0–5. In the distributed lag model, we used continuous *T*_max_, but for ease of comparison between our pre- and post-HAP years, we calculated the RR of mortality at each whole number *T*_max_ and across lag structures for each year. We used contour plots comparing the lagged temperature-mortality relationships to identify the most appropriate lag structures for subsequent analyses described below.

#### 2.5.3. Calculation of Incidence Rates and Incidence Rate Ratios

To generate estimates of absolute risk and feed into calculations of potential mortality avoided after HAP implementation, we also performed an analysis of daily mortality incidence for different heat alert categories. To generate crude incidence rate (IR) estimates, we used daily mortality count data and annual estimated population data to calculate IRs for each summer day in 2007–2010 and 2014–2015 using Excel. We stratified estimates by *T*_max_, grouping estimates by heat alert category (i.e., any alert and yellow, orange, or red alert, respectively) as described above. We used the Mann–Whitney *U* test to compute *p* values for differences between IRs. We also calculated incidence rate ratios (IRRs) comparing 2014 with 2010 and calculated 95% confidence intervals for both IRs and IRRs using methods described in Rothman et al. [36].

#### 2.5.4. Estimation of Temperature-Mortality Relationships for Pre- and Post-HAP Periods

Using pre-HAP IRs and counts of days in each warning category in the post-HAP period, we generated estimates of expected death counts for days in the post-HAP period when heat alerts were called. We subtracted observed mortality from these estimates to estimate avoided mortality post-HAP.

#### 2.5.5. Sensitivity Analysis

2010 was an anomalously hot year in the pre-HAP period, with substantial mortality associated with a record-breaking heat wave. We evaluated the sensitivity of the results to this anomalous event by performing the analyses without data from 2010.

### 2.6. Ethics Review

The research was reviewed and approved by the institutional review boards of our respective institutions.

### 2.7. Role of the Funding Sources

None of the funding sources had a role in study design; in data collection, analysis, or interpretation; in writing this report; or in the decision to submit this paper for publication.

## 3. Results

**Figure 1:**
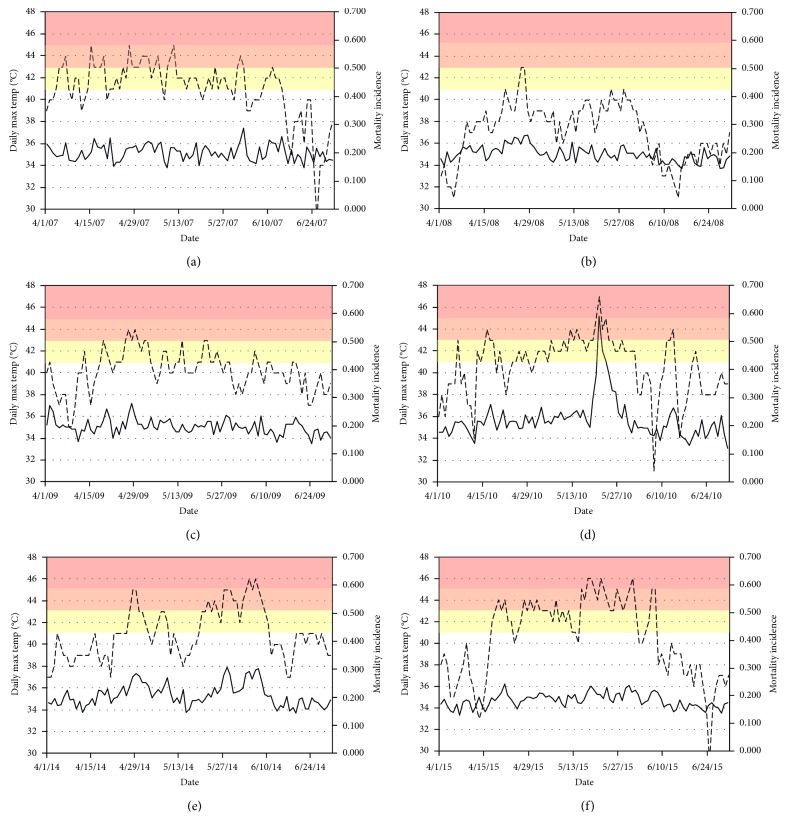
Pre-HAP (2007–2010) and Post-HAP (2014–2015) summer daily temperatures (dotted lines) and daily mortality incidence (solid lines). (a) Daily max temp and mortality 2007. (b) Daily max temp and mortality 2008. (c) Daily max temp and mortality 2009. (d) Daily max temp and mortality 2010. (e) Daily max temp and mortality 2014. (f) Daily max temp and mortality 2015.

**Figure 2:**
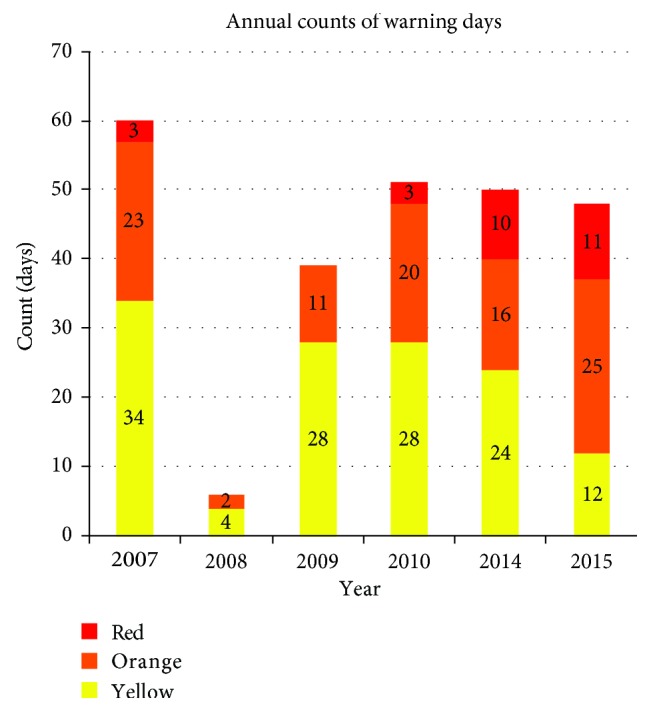
Annual counts of days with *T*_max_ in each category before HAP (2007–2010) and after HAP (2014–2015).

Compared with the pre-HAP period, the post-HAP period had a greater frequency of very hot (orange alert) and extremely hot (red alert) days, as evident in Figures 1 and 2. 2008 stands out as an unusually cool year in the sample studied.

**Figure 3:**
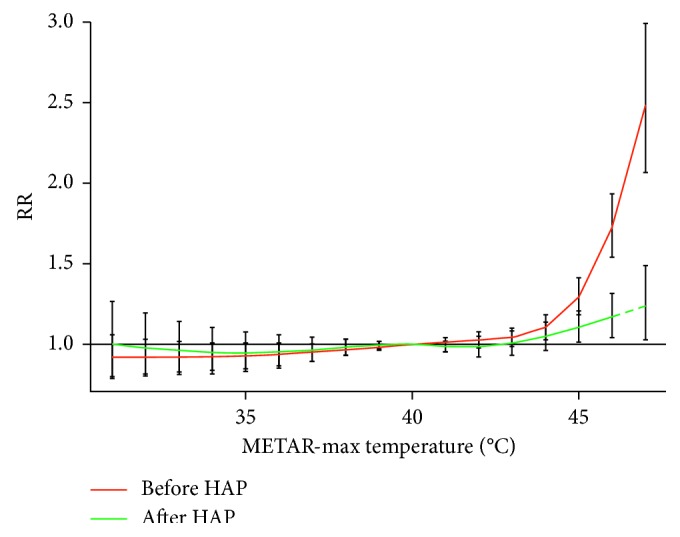
Results of distributed lag nonlinear model analysis. Red line indicates rate ratio (RR) for pre-HAP period, and green line indicates RR for post-HAP period (both relative to same-day T_max_ 40°C). Dashed green line shows the post-HAP RR estimated at 47°C.

The results of the distributed lag nonlinear model (dlnm) analysis are presented in Figure 3, which depicts the nonlagged, for example, lag 0, RR of mortality across the daily temperature strata for each year. The largest effect was seen at lag 0 for both years. Before HAP, the RR of mortality increased monotonically over 40°C with maximum effect (RR of 2.34; 95% CI 1.98–2.76) at 47°C, lag 0. After HAP, the RR also increased monotonically over 40°C, but with a substantially lower maximum effect (RR of 1.25; 1.02–1.53) estimated at 47°C, lag 0. Nonlagged estimates were used for the remaining analyses.

The results of the stratum-specific IRs for each period are presented in Table 1. IRs for both the pre- and post-HAP periods for each temperature category (i.e., any alert and yellow, orange, and red alerts, respectively) are presented. Consistent with the analysis of RR associated with extreme heat, mortality incidence was highest during the warmest periods, and there was a dose-response relationship observed in each period. After HAP, we observed a reduction in all-cause mortality incidence on all warning days during the post-HAP period, including days with any warning and days with yellow, orange, and red warnings, respectively. IRRs varied inversely with temperature, indicating the largest risk reduction on the hottest days. None of the differences were statistically significant.

Table 2 contains results from the expected mortality analysis. The counts of post-HAP days are presented for each category. Based on reductions in temperature-specific mortality incidence, an estimated 2,380 (95% CI 324–4,435) deaths were avoided in the post-HAP period, or an estimated 1,190 (95% CI 162–2,218) average annualized deaths avoided post-HAP.

In the sensitivity analysis in which 2010 data were removed from the pre-HAP period, the trend toward reduced mortality in the post-HAP period remained, but none of the differences was statistically significant (data not shown).

## 4. Discussion

Using two different approaches, we found a decrease in all-cause mortality in the first two years (2014–2015) after the HAP was implemented. The findings of this ecological study suggest that the HAP was associated with reduced mortality during the 2014–2015 heat seasons, particularly at higher temperatures. The maximum pre-HAP RR was 2.34 (95% CI 1.98–2.76) at 47°C (lag 0), and the maximum post-HAP RR was 1.25 (1.02–1.53) estimated at 47°C (lag 0). Post-to-pre-HAP nonlagged mortality IRR for *T*_max_ over 40°C was 0.95 (0.93–1.22) and 0.73 (0.29–1.81) for *T*_max_ over 45°C. An estimated 2,380 (95% CI 324–4,435) deaths were avoided in the post-HAP period. Temperatures and HAP warning days post-HAP implementation were associated with decreased summertime all-cause mortality rates, with the largest declines at the highest temperatures.

Ahmedabad's heat action plan, as detailed elsewhere [18], includes a suite of educational and other interventions, including a heat early warning system, that were new to the city and its population. The plan's messaging encourages the population to avoid heat exposure, maintain appropriate levels of hydration, and outlines strategies for cooling when becoming overheated. When heat warnings are triggered, a number of other activities occur, including actions to increase preparedness in the prehospital setting, clinics, and hospital emergency departments. Combined, these messages and activities may have reduced exposure and limited heat-related morbidity and mortality and may be a driver of the observed mortality reduction.

While encouraging, the findings should be interpreted with some caution, as other factors including heat wave timing and unmeasured factors may have affected the findings and several potential biases and confounders including air pollution were not controlled for in our analysis. Thus, we are not able to completely rule out other possible reasons for these findings. Population resilience to extreme heat may have increased for reasons unrelated to the heat action plan, for example, in response to publicity related to the 2010 heat wave. Alternatively, differences in the population or its exposure to ambient temperatures may be responsible. In 2010, for instance, the hottest days of the heat wave occurred slightly earlier in the season, when population susceptibility has been observed in Europe and North America to be approximately twice as high as later in the summer [37]. It is possible that the observed temperature-mortality associations were affected by other unmeasured factors, such as infectious disease outbreaks at certain points, though, as noted in our previous analysis of the 2010 heat wave, the principle source of data for pre-HAP exposure at very high temperatures, no such exposures were detected [26]. Demographic shifts or changes in living conditions (e.g., the prevalence of air conditioning, evaporative cooling, or crowded housing conditions) between the two periods may also have reduced underlying risk associated with temperature. Unfortunately, more granular data regarding population composition and changes over time are unavailable. Lastly, air pollution, an unmeasured potential confounder, may have had a role. Some work suggests that failure to control for ozone and particulates can result in an overestimation of heat-related mortality [38]. It is possible that Ahmedabad's air quality improved from 2007 to 2015 and that this drove some of the reduction, though such a reduction would have come during a period of rapid urban and industrial growth. Unfortunately, there are no air quality data to introduce as a control in our model.

The sensitivity analysis demonstrated that the findings depend on inclusion of data from 2010. This is likely because there were relatively few very hot days in the pre-HAP period from which exposure-outcome associations could be derived, limiting statistical power to detect a difference. Nevertheless, we cannot fully exclude the possibility that, as noted above, other factors may have confounded the relationships observed between temperature and mortality in 2010.

With all of these caveats in mind, we note that our findings are consistent with evaluations of similar heat preparedness initiatives adopted in other countries. Fouillet et al. compared the observed and expected mortalities during the 2006 heat wave in Europe based on heat wave of the summer of 2003 [39]. Their analysis showed 4,400 fewer deaths during the 18-day period (11^th^ to 28^th^ July) of the 2006 heat wave than expected based on the 2003 heat dose-response function, a reduction of 68%, more substantial than that observed in our study. The authors concluded that the mortality reduction could be due to increased public awareness, the promotion of preventive measures, and the establishment of an early warning system [40] after the 2003 heat wave in Europe.

Another ecological study of mortality and temperature, comparing two summers in Shanghai (1998 and 2003), found similar results, though the mortality reduction was less dramatic than that observed for Paris [41]. The authors generated a statistical model controlling for the effects of air pollution and temperature variation between the two years and observed fewer deaths during the 2003 heat wave, with an absolute reduction in mortality of approximately 29%. This reduction is slightly higher than we observed. The study authors concluded that the reduction likely resulted from the introduction of an early warning system, improved living conditions, and greater access to air conditioning [41].

Our findings are also generally compatible with those of the handful of other studies evaluating heat early warning system efficacy though methods are less directly comparable. One noted that extreme heat warnings reduced ischemic heart disease deaths among elderly in Hong Kong by 23% [42]. Another noted that a suite of changes including improvements in a municipal heat early warning system reduced expected deaths by at least 49% from 1995 to 1999 in the U.S. city of Milwaukee [43] A third in Florence, Italy, noted a roughly 9% decrease in odds of heat-related mortality among frail elderly after implementation of a heat early warning system [44]. Lastly, an econometric study of heat early warnings as part of a suite of disaster risk reduction strategies in Odisha, India, also found evidence of efficacy for including heat warnings as part of a larger disaster risk reduction effort [45]. None of these estimates is directly comparable but all of the studies provide evidence of risk reduction of a similar order of magnitude.

Our findings suggest that the Ahmedabad HAP protected health against mortality associated with extreme heat. Our ecological study design is limited and potentially subject to a number of biases, and we did not control for potential confounders including heat wave timing and air quality. An alternate design controlling for these factors and comparing associations between temperature and mortality in Ahmedabad and another city would add strength to the conclusions here, and additional research in Ahmedabad is ongoing. Furthermore, the analysis is limited by the small sample of very hot days across post-HAP study years (2014–2015) versus the baseline years (2007–2010), making it difficult to parse the data by including all other variables (e.g., day of week and changing age structure of municipal population) to find a significant HAP effect signal, even if additional data are available. That said, our pilot findings are consistent with recent reviews of heat action plans [29, 30], including the above studies, that heat warning systems save lives and their implementation in at-risk communities should be a priority for climate change adaptation.

Our findings suggest that other cities at risk for heat-related mortality in South Asia may benefit from Ahmedabad's example. The national government in India has created guidance based on the Ahmedabad HAP [46] and has worked to scale up heat early warning systems and action plans to 11 states and 30 cities in India, with several new HAPs being developed or now in place [47]. The Ahmedabad HAP, as well as the Indian government's scale-up effort, can serve as models for neighboring South Asian countries interested in increasing resilience to extreme heat.

## 5. Conclusions

In low- and middle-income countries, there are very few heat action plans and there is a lack of evidence of their effectiveness. Our study is one of the first to offer such evidence and suggests that the Ahmedabad intervention reduced mortality in its first two years, particularly at higher temperatures. Our results indicate that that heat action plans may be effective in protecting vulnerable populations in tropical latitudes, where the effect of climate change could be very severe on economically disadvantaged communities in crowded cities. While more research is needed, the results of this evaluation may encourage other cities and countries to develop similar efforts to reduce current risks and advance climate change adaptation.

## Figures and Tables

**Table 1:** All-cause mortality incidence rates (deaths per 100,000 population-years, with 95% CIs) for the pre- and post-HAP periods for the categories of any warning, yellow warning, orange warning, and red warning, respectively, as well as incidence rate ratios comparing post-HAP to pre-HAP IRs and 95% CIs.

Period	Any warning (>40°C)	Yellow warning (41–42.9°C)	Orange warning (43–44.9°C)	Red warning (≥45°C)
Pre-HAP IR (95% CI)	819 (788–850)	776 (753–798)	857 (830–883)	1244 (789–1699)
Post-HAP IR (95% CI)	775 (750–800)	708 (674–741)	773 (746–799)	908 (843–973)
IRR (95% CI)	0.95 (0.73–1.22)	0.91 (0.62–1.34)	0.90 (0.60–1.35)	0.73 (0.29–1.81)

**Table 2:** Counts of post-HAP days in each warning category and expected and observed mortality post-HAP and avoided mortality with 95% confidence intervals.

Variable	Any warning	Yellow warming	Orange warning	Red warning
Days Post-HAP	98	36	41	21
Expected deaths after HAP (95% CI)	16,012 (13,956–18,067)	5015 (4868–5162)	6307 (6114–6500)	4690 (2974–6406)
Observed deaths after HAP	13,632	4608	5634	3390
Avoided mortality after HAP (95% CI)	2380 (324–4435)	407 (260–554)	673 (480–886)	1300 (-416–3016)
Average annualized avoided mortality after HAP (95% CI)	1190 (162–2218)	203 (130–277)	336 (240–443)	650 (-208–1508)

## Data Availability

The data used in this analysis are available. Temperature and population data are available for download as described in Methods. Daily death counts are available on request from the corresponding author.
